# PRF Thermometry during MR-guided Focused Ultrasound Ablation in a Preclinical Thiel Model

**DOI:** 10.1186/2050-5736-2-S1-A11

**Published:** 2014-12-10

**Authors:** Ioannis Karakitsios, Andreas Melzer

**Affiliations:** 1 Institute for Medical Science and Technology (IMSaT), University of Dundee, Scotland, UK

## Background

MR guided Focused Ultrasound Surgery (MRgFUS) is a widely accepted treatment option that utilizes MR imaging for treatment planning, and Focused Ultrasound to ablate tumour and significantly reduce its size. Proton Resonance Frequency (PRF) Thermometry method is a useful tool for treatment planning, as it provides with accurate, real-time temperature maps. The aim of the present study was to determine the accuracy PRF Thermometry during MRgFUS on explanted Thiel embalmed human and animal liver, fresh animal liver, and compare to polyacrylamide gel phantom.

## Materials and methods

PRF Thermometry was conducted on 1.5T MRI system (Signa HDx, GE Medical Systems, Milwaukee, WI, USA) with embedded FUS table (ExAblate 2000 Uterine Fibroid System, InSightec Ltd., Tirat Carmel, Israel). The phantom and the organs were sonicated for 20 seconds using the following acoustic protocols: 300J, 600J, 1000J and 1400J. The temperature rise which based on PRF Thermometry during sonication was compared to the actual temperature increase in the same conditions measured by fibre optic thermocouples.

## Results

For the Thiel embalmed organs, we found temperature difference varied from 1.17°C to 3.13°C for the ovine liver, and from 1.30°C to 3.10°C for the human liver. The treatment of both fresh animal liver and phantom resulted in temperature differences lower 0.40°C.

## Conclusion

Our results indicated that PRF Thermometry is accurate on fresh animal tissue, as the temperature differences measured in the fresh organ were low and similar to those found for the gel phantom, and this was in accordance with published studies. However, the temperature differences found in the Thiel embalmed organs were higher than the fresh organ. This shows that the Thiel embalming affects temperature distribution in the organ. Thus, PRF-based temperature calibration of the Focused Ultrasound machine for Thiel embalmed tissue is highly desirable. However, the similarity of temperature difference between the Thiel human and Thiel animal organs could facilitate the utilization of Thiel animal organs for studies involving temperature monitoring during MRgFUS ablations.

